# Probiotics VSL#3 Protect against Development of Visceral Pain in Murine Model of Irritable Bowel Syndrome

**DOI:** 10.1371/journal.pone.0063893

**Published:** 2013-05-15

**Authors:** Eleonora Distrutti, Sabrina Cipriani, Andrea Mencarelli, Barbara Renga, Stefano Fiorucci

**Affiliations:** 1 S.C. di Gastroenterologia ed Epatologia, Azienda Ospedaliera di Perugia, Perugia, Italy; 2 Dipartimento di Medicina Clinica e Sperimentale, Università degli Studi di Perugia, Perugia, Italy; University of California, Los Angeles, United States of America

## Abstract

**Background and Aims:**

Irritable bowel syndrome (IBS) is linked to post-inflammatory and stress-correlated factors that cause changes in the perception of visceral events. Probiotic bacteria may be effective in treating IBS symptoms. Here, we have investigated whether early life administration of VSL#3, a mixture of 8 probiotic bacteria strains, protects against development of visceral hypersensitivity driven by neonatal maternal separation (NMS), a rat model of IBS.

**Methods:**

Male NMS pups were treated orally with placebo or VSL#3 from days 3 to 60, while normal, not separated rats were used as controls. After 60 days from birth, perception of painful sensation induced by colorectal distension (CRD) was measured by assessing the abdominal withdrawal reflex (score 0–4). The colonic gene expression was assessed by using the Agilent Whole Rat Genome Oligo Microarrays platform and confirmed by real time PCR.

**Results:**

NMS rats exhibited both hyperalgesia and allodynia when compared to control rats. VSL#3 had a potent analgesic effect on CRD-induced pain without changing the colorectal compliance. The microarray analysis demonstrated that NMS induces a robust change in the expression of subsets of genes (CCL2, NOS3, THP1, NTRK1, CCR2, BDRKRB1, IL-10, TNFRSF1B, TRPV4, CNR1 and OPRL1) involved in pain transmission and inflammation. TPH1, tryptophan hydroxylase 1, a validated target gene in IBS treatment, was markedly upregulated by NMS and this effect was reversed by VSL#3 intervention.

**Conclusions:**

Early life administration of VSL#3 reduces visceral pain perception in a model of IBS and resets colonic expression of subsets of genes mediating pain and inflammation.

**Transcript profiling:**

Accession number of repository for expression microarray data is GSE38942 (http://www.ncbi.nlm.nih.gov/geo/query/acc.cgi?acc=GSE38942).

## Introduction

Irritable bowel syndrome (IBS) is a disorder characterized by chronic abdominal pain and discomfort associated with alterations in bowel habits in the absence of a demonstrable pathology [1]. Alterations in bowel habits are likely related to dysregulation of autonomic system in the gut, whereas symptoms of abdominal pain and discomfort are thought to involve additional changes in the perception of visceral events, in the form of hyperalgesia or allodynia [2]–[3]. Evidence is growing to support the notion that IBS might be a post-inflammatory and stress-correlated condition [4]–[5] and chronic gut inflammatory processes are thought to play a role in its pathogenesis.

The neonatal maternal separation model (NMS) is an early life stress experience that resets the expression of neurotransmitters, receptors and neurotransporters in the central nervous system (CNS) and predisposes adult rats to develop hyperalgesia to nociceptive visceral stimuli [6]–[9]. The altered physiological responses and visceral hyperalgesia of NMS rats are consistent with changes observed in IBS patients [6] making the NMS model a useful tool to investigate the pathophysiological mechanism of visceral hypersensitivity in functional gastroenterological disorders [10].

The intestinal microbiota plays essential roles in nutrient absorption and metabolism, immune stimulation, satiety and pain. An altered composition of intestinal microbiota has been reported in IBS patients [11]–[12] while its modification by probiotic diet reduces visceral hypersensitivity in experimental models of abdominal pain by modulating neural functions [13]–[16]. The probiotic VSL#3 is a mixture of 8 Gram-positive bacteria strains [17]. VSL#3 improves the outcome of patients with chronic intestinal inflammation [18]–[22], ameliorates abdominal bloating [23], reduces flatulence scores and delays colonic transit without altering bowel function in IBS patients [24] and children [25]. Nevertheless, the mechanism that underlies the beneficial effects of VSL#3 in these settings is still poorly defined.

In the present study we have made an attempt to identify mechanisms involved in beneficial effects exerted by VSL#3 intervention in a model of visceral hypersensitivity induced in rats by NMS by using a global gene expression analysis. Our results indicates that NMS causes both allodynia and hyperalgesia and influences the expression of a wide array of genes, including genes known to mediates inflammation and pain. Our results also demonstrate that VSL#3 intervention was effective in both reverting NMS-induced visceral hypersensitivity and resetting the complex network of genes involved in inflammation and pain.

## Materials and Methods

### Animals

Male, Wistar rats (200–250 g, Charles River, Monza, Italy) were housed in plastic cages and maintained under controlled conditions with 12-hour light/dark cycles (lights on at 07.00). Tap water and standard laboratory chow were freely available. Food was withheld for 12 hours before CRD recordings. All the animals were individually trained by spending 2–3 hours per day in a Plexiglas cage for 2–3 days. This allowed them to adjust to a movement-restriction environment similar to that adopted during the distending procedure. All experimental procedures described below were approved by the institutional animal research committees of University of Perugia (Permit Number: 98/2010-B) and were in accordance with nationally approved guidelines for the treatment of laboratory animals. All experiments were performed in conscious rats and conducted in a blind manner in that the observers were not aware of the identity of drugs administered to each animal.

### The Neonatal Maternal Separation Model

A neonatal maternal separation (NMS)-induced visceral hyperalgesia rat model has been previously established [6]. Because its characteristics mimic the symptoms of IBS patients, it is often used to study the mechanism of visceral hyperalgesia and to evaluate the pharmacological effects of potential IBS therapies [10], [26]. Briefly, pups in the NMS group were separated from their mothers and placed into individual cages in another room 180 min daily from postnatal day 2 to day 14, whereas normally-handled (NH) pups remained undisturbed in their home cage with the dam. All pups were weaned on postnatal day 22, and only male pups were used in the present study to avoid hormonal cycle induced variations. Male rats on postnatal day 60 were used in a series of CRD experiments.

### Experimental Design - Effects of VSL#3 on Colonic Nociception and Compliance

Rats were divided in 7 groups of 5 animals each. All rats, except those of Group 1 (healthy, intact) and Group 3 (NMS, intact), were treated daily by gavage with placebo or VSL#3 at the dose of 17 billions in 100 µL saline according to the experimental design (Table 1) [27]. Placebo was administered only to animals of Group 2 that corresponds to healthy animals in which CRD was performed and Group 4 that corresponds to NMS rats in which CRD was performed. Group 5, Group 6 and Group 7 included NMS rats treated with VSL#3 from day 3 to day 60, from 3 to day 15 and from day 45 to day 60 respectively (Table 1). Microarray studies were conducted only in Group 2 (healthy, CRD, named Group C), Group 4 (NMS, CRD, named Group M) and Group 5 (NMS, VSL#3 administered from day 3 to day 60, CRD, named Group D). At the end of the studies, animals were sacrified and colon, blood and spinal cord collected for further determinations.

**Table 1:** Experimental design.

Name of the group	Abbreviation	Treatment
Normal group	Group 1	Healthy, intact
Control group	Group 2, named Group C*	Healthy, placebo, CRD
NMS group	Group 3	NMS, intact
Neonatal-Maternal separation, NMS group	Group 4, named Group M*	NMS, placebo, CRD
Probiotic Diet, NMS+VSL#3 (day 3–60) group	Group 5, named Group D*	NMS, VSL#3 from day 3 to day 60, CRD
NMS+VSL#3 (day 3–15) group	Group 6	NMS, VSL#3 from day 3 to day 15, CRD
NMS+VSL#3 (day 45–60) group	Group 7	NMS, VSL#3 from day 45 to day 60, CRD

* Groups in which microarray analysis was performed.

### CRD and Behavioral Testing

The distending protocol was performed as previously described [28]–[32]. The night before CRD experiments, the balloons (7–8 mm diameter) were inflated and left overnight so that the latex stretched and the balloons became compliant. On the testing day, each rat was sedated with ether inhalation and the latex balloon (1.5 cm long) and the probe catheter (0.5 cm) was inserted intrarectally and fixed at the base of the tail. The balloon was connected via a double barreled cannula to a pressure transducer to continuously monitoring the colorectal pressure by a computer (PowerLab PC, A.D. Instruments, Milford, MA, USA) and to a syringe for inflation/deflation of the balloon. The rats were then housed in a small Plexiglas cage (20×8×8 cm) on an elevated platform and allowed to regain consciousness and adapted for 1 hour. After recovery from sedation, the rats underwent the CRD procedure and behavioral response was tested in all groups except groups 1 and 3 in which no CRD was performed. Infusion of water was performed by hands. CRD of 20 seconds performed every 5 minutes was applied in increment of 0.4 ml starting from 0.4 ml and increasing to 1.6 ml water. To achieve an accurate measurement of the colonic parameters and perception, each distension was repeated twice and data were averaged for analysis. Behavioral responses and colonic parameters collected during the first and the second sets of CRD were assessed and compared among all groups [28]–[32]. The behavioral response to CRD was assessed by measuring the abdominal withdrawal reflex (AWR) using a semiquantitative scoring system [33]–[34]. The AWR is an involuntary motor reflex similar to the visceromotor reflex, but it has the great advantage that the latter requires abdominal surgery to implant recording electrodes and wires in the abdominal muscle wall, which may cause additional sensitization [34]. Measurement of the AWR consists of visual observation of the rat’s response to graded CRD by a blinded observer and assignment of an AWR score according with the behavioral scale previously described [33], in which grade 0 corresponds to no behavioral response to CRD, grade 1 corresponds to brief head movement at the onset of the stimulus followed by immobility, grade 2 corresponds to a mild contraction of abdominal muscles although the rat does not lift the abdomen off the platform, grade 3 corresponds to a strong contraction of the abdominal muscles with the lifting of the abdomen off the platform, and grade 4 corresponds to a severe contraction of the abdominal muscles manifested by body arching and the lifting of the abdomen and of the pelvic structures and scrotum. The rats that did not show any behavioral response (i.e. score 0) were excluded. To determine the effect of placebo or VSL#3 on colonic smooth muscle, the compliance of the colon during CRD was obtained from colorectal volume and pressure and expressed as ml/mmHg [28]–[32].

### Microarray Analysis

Microarray analysis was performed on colonic samples from Group C, Group M and Group D. The same segment was taken in each animal starting approximaly 2 cm from the anus. The data discussed in this publication have been deposited in NCBI’s Gene Expression Omnibus (GEO) and are accessible through GEO Series accession number GSE38942 (http://www.ncbi.nlm.nih.gov/geo/query/acc.cgi?acc=GSE38942) [35]–[36].

All microarray analysis were performed by Miltenyi Biotec, GmbH Bioinformatics, German.

#### RNA extraction

The RNA was isolated from rat tissue samples by using standard RNA extraction protocols (Trizol), the RNA quality-checked via the Agilent 2100 Bioanalyzer platform (Agilent Technologies) from the following treatment groups:

Group C = healthy rats+CRD

Group M = NMS rats+CRD

Group D = NMS rats+probiotic Diet from day 3 to day 60+ CRD

For each condition, four biological replicates exist.

#### Linear T7-based amplification of RNA

For the linear T7-based amplification step, 100 ng of each total RNA sample was used. To produce Cy3-labeled cRNA, the RNA samples were amplified and labeled using the Agilent Low Input Quick Amp Labeling Kit (Agilent Technologies) following the manufacturer’s protocol. Yields of cRNA and the dye-incorporation rate were measured with the ND-1000 Spectrophotometer (NanoDrop Technologies).

#### Hybridization of agilent whole genome oligo microarrays

The hybridization procedure was performed according to the Agilent 60-mer oligo microarray processing protocol using the Agilent Gene Expression Hybridization Kit (Agilent Technologies). Briefly, 0.6 µg Cy3-labeled fragmented cRNA in hybridization buffer was hybridized overnight (17 hours, 65°C) to Agilent Whole Rat Genome Oligo Microarrays 8×60 K using Agilent’s recommended hybridization chamber and oven.

#### Scanning results

Fluorescence signals of the hybridized Agilent Microarrays were detected using Agilent’s Microarray Scanner System (Agilent Technologies).

#### Image and data analysis

The Agilent Feature Extraction Software (FES) was used to read out and process the microarray image files. The software determines feature intensities (including background subtraction), rejects outliers and calculates statistical confidences. For determination of differential gene expression FES derived output data files were further analyzed using the Rosetta Resolver gene expression data analysis system (Rosetta Biosoftware). All samples were labelled with Cy3, here, the ratio experiments are designated as control versus (vs) sample experiments (automated data output of the Resolver system).

The ratios are always calculated by dividing sample signal intensity through control signal intensity.

The bioinformatics data analysis of eleven microarray datasets obtained from one-color hybridization of rat RNAs on Agilent Whole Rat Genome Oligo Microarrays 8×60 K was performed.

As the aim of the study was to identify differentially expressed genes in the comparisons between all conditions, the differentially expressed genes were further filtered for functional associations related to neurotransmitters/mediators of pain, as well as for associations with cytokines/immunity and inflammatory responses.

The data was processed as follows:

1 Preprocessing of the data, including normalization and correlation analysis
2 Differential gene expression analysis (DGA) for the following groups:
  - Group M versus Group C
  - Group D versus Group M
  - Group D versus Group C

The analyses aim at distinguishing expression changes between all groups of samples so that six discriminatory gene sets (for each group up- and downregulated genes) were analyzed. A combination of statistical methods and the magnitude of expression difference (fold change) were applied in order to identify genes with differential expression between two sample groups. For the detection of discriminatory expression, genes had been selected that show a statistically significant deviation in the test compared to the reference group (ANOVA p-value ≤0.05, tukey p≤0.05). At the same time, it was required that the average expression value was at least 2-fold higher or lower than the reference average. For allowing a visually appealing display as a red/green heatmap, the expression values were converted to “virtual ratios” by referencing each individual intensity signal to median of all intensities. The base-2 logarithms of these virtual ratios were used for heatmap display. In each heat map the lanes from C1 to C4 corresponded to 4 rats of Group C, the lanes from M5 to M8 corresponded to 4 rats of Group M, and the lanes from D9 to D11 corresponded to 3 rats of Group D. A comparison of the discriminatory gene sets among different groups was showed by using the Venn diagrams.

3 Functional grouping analysis, including custom bioinformatics to identify associations of differentially expressed genes obtained in any of the DGAs with:
  - neurotransmitters and/or mediators of pain
  - cytokines, immunity, and inflammatory responses.

The functional grouping and annotation analysis provides an overview of the different biological processes and pathways, which are modulated in the discriminatory analyses. Here, the reporters were annotated with information from various databases in order to find common features among the genes sharing similar expression characteristics. The annotations used were derived from Gene Ontology (GO), which provides information on molecular function, as well as various pathway resources for information on involvement in biological signalling pathways. In addition, the gene lists obtained were also compared to known targets of certain signaling pathways. The significantly modified genes were grouped into 10 principal pathways, namely Cellular behavior, Cellular process, Metabolism, Organismal, Signaling, Signaling/mechanism, Stress, Structural, Pain, Inflammation. GO classification separated the genes involved in different biological processes, molecular functions and cellular components taking into account that the same gene could be present in more than one pathway. The annotation analysis was then completed by representative bar charts that give an overview of the biological categories found most frequently among the genes of the input reporter set. As the number of genes in the categories varies considerably, the size of the bars does not indicate a particular biological importance or over-representation.

4 Interaction network: core inflammatory pathway

The observations made in previous analyses were confirmed in the interaction graphs of key regulatory molecules of the core inflammatory pathways generated with Cytoscape (www.cytoscape.org).

### PCR Analysis

Quantification of the expression of selected genes was performed by quantitative real-time PCR (qRT-PCR). 1 µl of the remaining RNA from colon samples that were used for gene array was incubated with DNase I and reverse-transcribed with Superscript II (Invitrogen) according to manufacturer specifications. For real-time PCR, 1 µl of template was used in a 25-µl reaction containing a 0.2 µM concentration of each primer and 12.5 µl of 2× SYBR Green PCR Master Mix (Bio-Rad Laboratories, Hercules, CA). All reactions were performed in duplicate using the following cycling conditions: 2 min at 95°C, followed by 50 cycles of 95°C for 10 s and 60°C for 30 s using an iCycler iQ instrument (Bio-Rad Laboratories). The mean value of the duplicates for each sample was calculated and expressed as cycle threshold (C_T_). The amount of gene expression was then calculated as the difference (ΔC_T_) between the C_T_ value of the sample for the target gene and the mean C_T_ value of that sample for the endogenous control (GAPDH). Relative expression was calculated as the difference (ΔΔC_T_) between the ΔC_T_ values of the test and control samples for each target gene. The relative level of expression was measured as 2^−ΔΔCT^. All PCR primers were designed using the software PRIMER3-OUTPUT using published sequence data obtained from the NCBI database (Table 2).

**Table 2:** Sense and antisense probes for genes related to “Pain” annotation.

Oligo Name	Sense	Antisense
rCCL2	atgcagttaatgccccactc	ttccttattggggtcagcac
rNOS3	caatcttcgttcagccatca	gggtccagccatgttgaata
rNTRK1	gtctggtgggtcagggacta	cacacatcactctcggtgct
rTPH1	gacatctttcccctgctgaa	tctttgaagccaggatggtc
rCCR2	ctgcccctacttgtcatggt	ggcctggtctaagtgcatgt
rBDKRB1	ccccgtgactgctatcatct	agaccaggaaggaggctacc
rIL10	ggagtgaagaccagcaaagg	ggcaacccaagtaaccctta
rTNFRSF1B	ggctcagatgtgctgtgcta	atgcagatggttccagacct
rTRPV4	cgatatgaggcgacaggact	gggagcacttgagaagcaac
rCNR1	agagcatcatcatccacacg	tcaacaccaccaggatcaga
rOPLR1	aagagatcgagtgcctggtg	agcacagggatgatgaagga

### Statistical Analysis

Behavioral data are presented as mean ± SE, with sample sizes of at least 5 rats per group. Statistical comparisons were performed by the Mann-Whitney test for unpaired data and by the Wilcoxon signed rank test for paired data when two group of data were analyzed, and by the ANOVA for non parametric data, Kruskal-Wallis followed by Dunns comparison of selected pairs of column, when more than two groups of data were analyzed. An associated probability (p value) of less that 5% was considered significant.

For microarray analysis a combination of statistical methods (ANOVA p-value ≤0.05, tukey p≤0.05) and the magnitude of expression difference (2-fold change higher or lower than the reference average) was applied. For allowing a visually appealing display as a red/green heatmap, the expression values were converted to “virtual ratios” by referencing each individual intensity signal to median of all intensities. The base-2 logarithms of these virtual ratios were used for heatmap display in which lanes from 1 to 4 correspond to control rats, lanes from 5 to 8 correspond to NMS animals and lanes from 9 to 11 correspond to NMS rats treated with probiotic diet (VSL#3 3–60 day). A comparison of the discriminatory gene sets among different groups was performed by using the Venn diagrams.

## Results

### VSL#3 Reverses Both Allodynia and Hyperalgesia in NMS Rats

**Figure 1:**
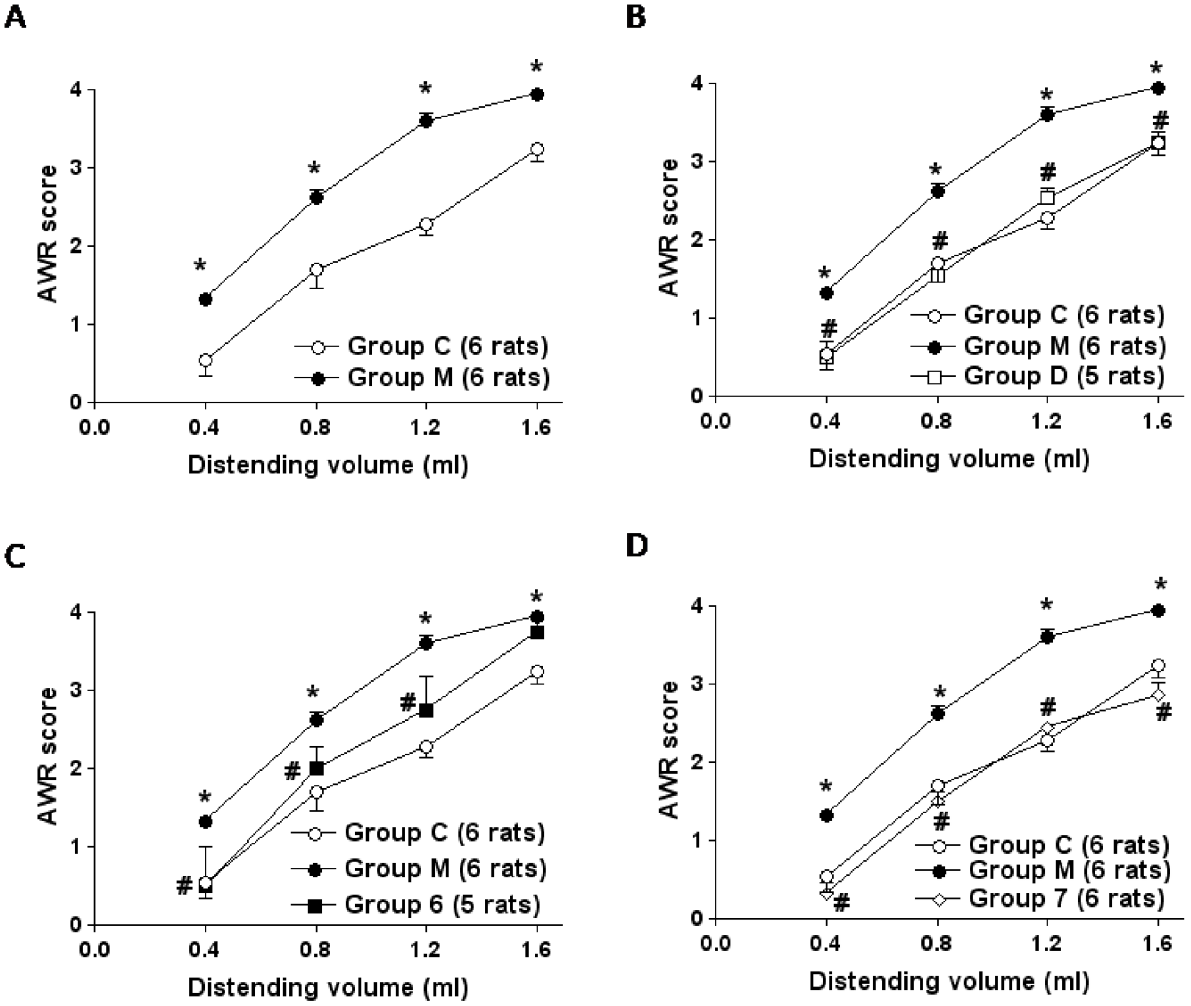
VSL#3 reverses hypersensitivity induced by NMS. (A) Group C and Group M are the same in all comparison experiments. In healthy animals (Group C, 6 rats) CRD (0.4–1.6 ml water) elicited a volume-dependent increase of the AWR scores while caused allodynia and hyperalgesia in NMS animals (Group M, 6 rats). (B, D) VSL#3 administered from day 3 to day 60 (Group D, 5 rats) and from day 45 to day 60 (Group 7, 6 rats) restored normal sensitivity. (C) Probiotic intervention from day 3 to day 15 (Group 6, 5 rats) was only partially effective in visceral sensitivity as hyperalgesia persisted. Statistical comparisons were performed by the Mann-Whitney test for unpaired data and by the Wilcoxon signed rank test for paired data when two group of data were analyzed, and by the ANOVA for non parametric data, Kruskal-Wallis followed by Dunns comparison of selected pairs of column, when more than two groups of data were analyzed. *p<0.05 vs Group C; ^#^p<0.05 vs Group M.

In all experimental settings, animals were awake and no changes in the consciousness state were produced by CRD and VSL#3 intervention. For each CRD, two sequential distension-effect curves were constructed and the two obtained scores were averaged. In control animals (Group C), CRD (0.4–1.6 ml water) elicited a volume-dependent increase of the AWR scores which was rapid in onset, persisted for the duration of the distension period (Figure 1A) and returned to the baseline immediately after the distension was stopped. In the NMS animals (Group M), CRD induced both allodynia and hyperalgesia (Figure 1A). In NMS animals, VSL#3 administered from day 3 to day 60 (Group D) or from day 45 to day 60 (Group 7) caused the complete restoration of normal sensitivity (Figure 1B and D respectively), while probiotic intervention from day 3 to day 15 (Group 6) was only partially effective in reducing the visceral hypersensitivity (Figure 1C), as hyperalgesia persisted. *p<0.05 versus Group C; #p<0.05 versus Group M. Neither maternal deprivation and VSL#3 intervention had any effect on colorectal compliance (Figure S1).

### Effect of NMS and VSL#3 Treatment on Gene Expression

#### Global microarray analysis

**Figure 2:**
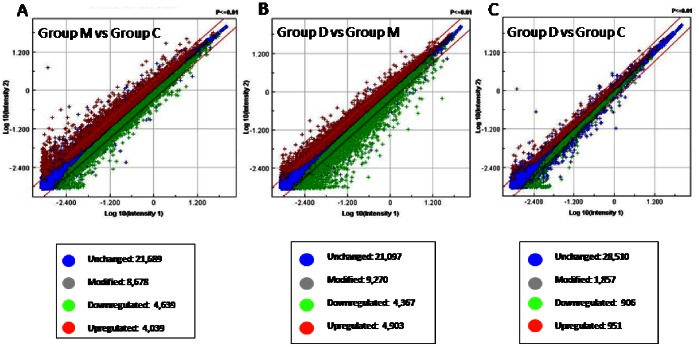
VSL#3 reverses the NMS-induced alteration of gene expression: global microarray analysis. (A) Expression of 8,678 (28.57% of total) genes was globally modified in NMS animals compared with normal rats (Group M vs Group C): 4,039 (13.29%) were upregulated and 4,639 (15.27%) were downregulated. (B) VSL#3 effectively resected gene expression in NMS rats (Group D vs Group M): 9,270 (30.5% of total) genes were globally modified, 4,903 (16.14 of total%) were upregulated, while 4,367 (14.38% of total) were downregulated. (C) VSL#3 administration to NMS rats caused a pattern of gene expression that was similar to that of control rats (Group D vs Group C). Microarray data from 3–4 replicates.

Global microarray analysis, performed on colonic tissue from groups C, M and D, detected several differentially expressed genes (data are available at the website http://www.ncbi.nlm.nih.gov/geo/query/acc.cgi?acc=GSE38942). On a total of 30,367 genes, expression of 8,678 (28.57%) genes was modified in NMS in comparison with healthy rats, 4,039 of which were upregulated and 4,639 downregulated (Figure 2A). Treating NMS rats with VSL#3 caused changes in the expression of 9,270 genes (30.5% of total): 4,903 genes were upregulated and 4,367 downregulated (Figure 2B). VSL#3 almost completely restored the initial pattern of gene expression in NMS rats. Thus, only 1,857 genes were modified in group D when compared with control rats, corresponding to 6.11% of total genes, with slight differentiation between up- and downregulation (51.22% and 48.78% respectively) (Figure 2C).

#### DGA and functional analysis

**Figure 3:**
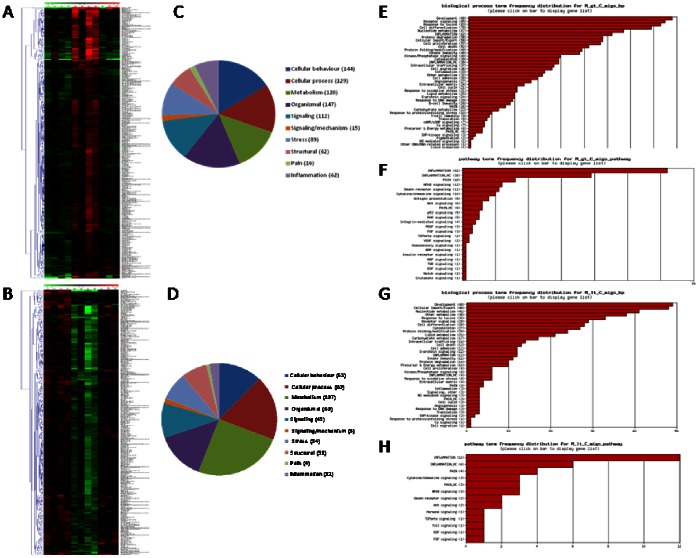
DGA and functional analysis of the effect of NMS on gene expression. (A–B) Heat maps of the significant genes (p<0.05 and 2-fold up- or downregulation) in NMS rats (Group M, lanes M5–M8) in comparison to VSL#3 treated NMS rats (Group C, lanes C1–C4). In comparison to control rats, NMS modulated the colonic expression of 665 genes (2% of total), 353 were upregulated and 312 downregulated. The expression of the majority of these genes returned to normal value after VSL#3 administration (Group D, lanes D9–D11). (C–D) Pie charts representing biological processes, molecular functions and cellular components among differentially expressed genes (up- or downregulation respectively). (E–G) Frequency distribution of the annotations for upregulated and downregulated genes respectively. (F–H) Frequency distribution of the signaling pathways for upregulated and downregulated genes respectively. gt: greater than; lt: lower than; migo_bp: gene ontology (GO) curated by Miltenyi Bioinformatics biological processs/function; migo_pathways: gene ontology (GO) curated by Miltenyi Bioinformatics pathways; Inflammation_HC: list of genes associated with inflammation from GO data but with more stringent selection criteria; Pain_HC: list of genes associated with pain from GO data but with more stringent selection criteria. Microarray data from 3–4 replicates.

When the combination of statistical methods (p-value <0.05) and the magnitude of gene expression difference (fold change at least ±2) was applied, 665 gene (2.18% of total) were significantly modulated in NMS animals compared to naïve rats (Table 3). The hierarchical clustering of these genes provided good separation based on the expression of the genes in the three groups of animals. In Figure 3A and B, red and green colours indicate upregulated and downregulated genes respectively, while black colour indicates no significant changes in gene expression. When NMS animals (lanes M5–M9) were compared with naive rats (lanes C1–C4), we detected a significant up- and downregulation of 353 and 312 genes respectively (Figure 3A and B). Moreover, the expression of these genes in group M was almost completely reverted by VSL#3 intervention (lanes D9–D11) (Figure 3A and B). Genes differentially expressed in NMS animals were classified according to their putative Gene Ontology (GO) based on significant similarity with known genes recorded in public databases (Figure 3C and D for up- and downregulated genes respectively). The frequency distribution of significantly modified genes in representative GO-derivative biological processes/functions (migo_bp) (Figure 3E and G for up- and downregulated genes respectively) and GO-derivative biological pathways (migo_pathways) (Figure 3F and H for up- and downregulated genes respectively) for group M vs group C is also illustrated. The up- and downregulated genes specifically involved in pain transmission are illustrated in Table 4 and Table 5 respectively, while the lists of genes involved in inflammation are available in the GEO system at the website http://www.ncbi.nlm.nih.gov/geo/query/acc.cgi?acc=GSE38942. Interestingly the number of upregulated genes was higher than that of downregulated genes in “Pain” and “Inflammation” categories, as only 4 and 12 genes were downregulated, while 15 (tryptophan hydroxylase 1 gene was identified by two different primers) and 62 genes were upregulated in the two functional annotations, respectively. Most notable among those genes known for their role in mediating pain in both humans and rodents, were tryptophan hydroxylase 1 (TPH1), chemokine [C–C motif] ligand 2 (CCL2), chemokine [C–C motif] receptor 2 (CCR2), bradykinin receptor B1 (BDKRB1), tumor necrosis factor receptor superfamily member 1b (TNFRSF1B), prostaglandin E receptor 2 (subtype EP2) (PTGER2), nitric oxide synthase 3 endothelial cell (NOS3), neurotrophic tyrosine kinase receptor type 1 (NTRK1), interleukin 6 (IL6) and transient receptor potential cation channel subfamily V members 4 and 2 (TRPV4 and TRPV2) (Table 4). Additionally, migo_bp and migo_pathways identified several upregulated genes also associated with immune and inflammatory responses (http://www.ncbi.nlm.nih.gov/geo/query/acc.cgi?acc=GSE38942).

**Table 3:** Number of genes that were modified ±2 folds (up- or downregulation) in different experimental groups.

Groups	Number of significantly modifiedgenes (% of total)	Number of significanly upregulated genes (% of total)	Number of significantly downregulated genes (% of total)
Group M vs Group C	665/30,367 (2.18)	353/30,367 (1.16)	312/30,367 (1.02)
Group D vs Group M	779/30,367 (2.56)	411/30,367 (1.35)	368/30,367 (1.21)
Group D vs Group C	108/30,367 (0.35)	64/30,367 (0.21)	44/30,367 (0.14)

**Table 4:** Upregulated genes specifically involved in pain transmission in NMS rats in comparison with normal animals.

Identifier	Short name	Long name
A_42_P47339	CXCL1	chemokine (C-X-C motif) ligand 1 (melanoma growth stimulating activity, alpha)
A_42_P695401	CCL2	chemokine (C-C motif) ligand 2
A_43_P12508	PTGER2	prostaglandin E receptor 2 (subtype EP2)
A_44_P198620	NOS3	nitric oxide synthase 3, endothelial cell
A_44_P306204	TPH1	tryptophan hydroxylase 1*
A_44_P371339	IL6	interleukin 6
A_44_P430547	NTRK1	neurotrophic tyrosine kinase, receptor, type 1
A_64_P048210	TPH1	tryptophan hydroxylase 1*
A_64_P057941	MRGPRG	MAS-related GPR, member G
A_64_P100793	CCR2	chemokine (C-C motif) receptor 2
A_64_P118628	BDKRB1	bradykinin receptor B1
A_64_P122382	IL10	Interleukin 10
A_64_P125973	TNFRSF1B	tumor necrosis factor receptor superfamily, member 1b
A_64_P129316	PTGS2	prostaglandin-endoperoxide synthase 2
A_64_P130174	TRPV4	transient receptor potential cation channel, subfamily V, member 4
A_64_P130184	TRPV2	transient receptor potential cation channel, subfamily V, member 2

* Two different probe sequences are used for the same gene on the array.

**Table 5:** Downregulated genes specifically involved in pain transmission in NMS rats in comparison with normal animals.

Identifier	Short name	Long name
A_44_P228891	FAAH	fatty acid amide hydrolase
A_44_P472874	TRPA1	transient receptor potential cation channel, subfamily A, member 1
A_44_P484909	P2RX4	purinergic receptor P2X, ligand-gated ion channel 4
A_64_P109894	P2RX4	angiotensin II receptor, type 1b

**Figure 4:**
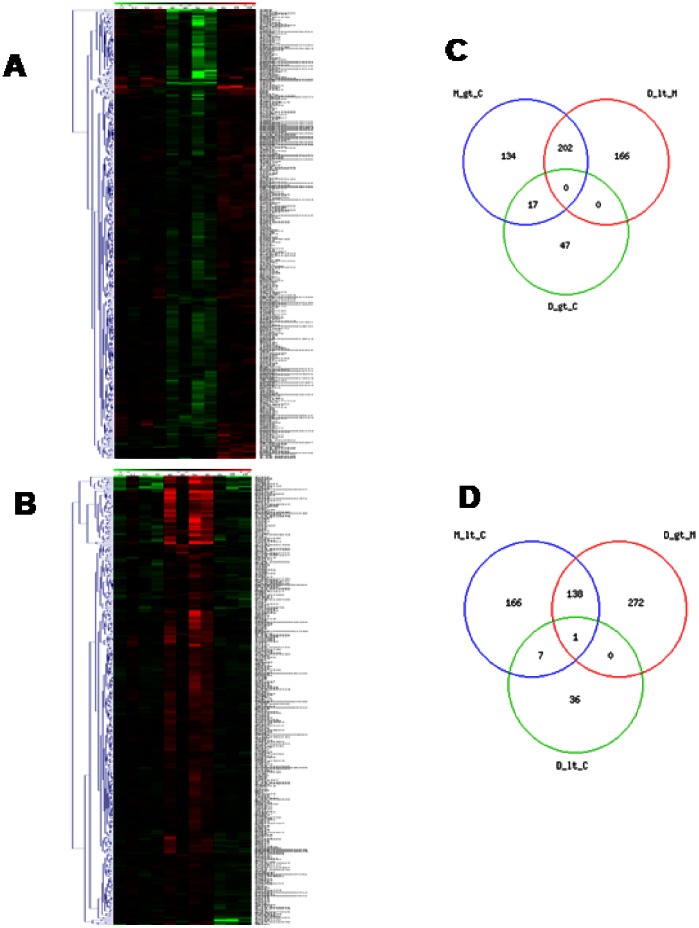
DGA and Venn diagrams of the effect of VSL#3 on NMS-induced alteration of gene expression. (A–B) Heat maps analysis of changes caused by VSL#3 administration (Group D, lanes D9–D11) to NMS rats (group M, lanes M5–M8) on genes whose expression was modulated >2-folds (up- or downregulation, p<0.05). (C–D) Venn diagrams of modulated genes. NMS induced an upregulation of 353 genes, 202 of which are downregulated by the probiotic treatment while 312 genes were dowunregulated, 139 of which are upregulated by VSL#3 administration. Microarray data from 3–4 replicates. gt: greater than; lt: lower than.

**Figure 5:**
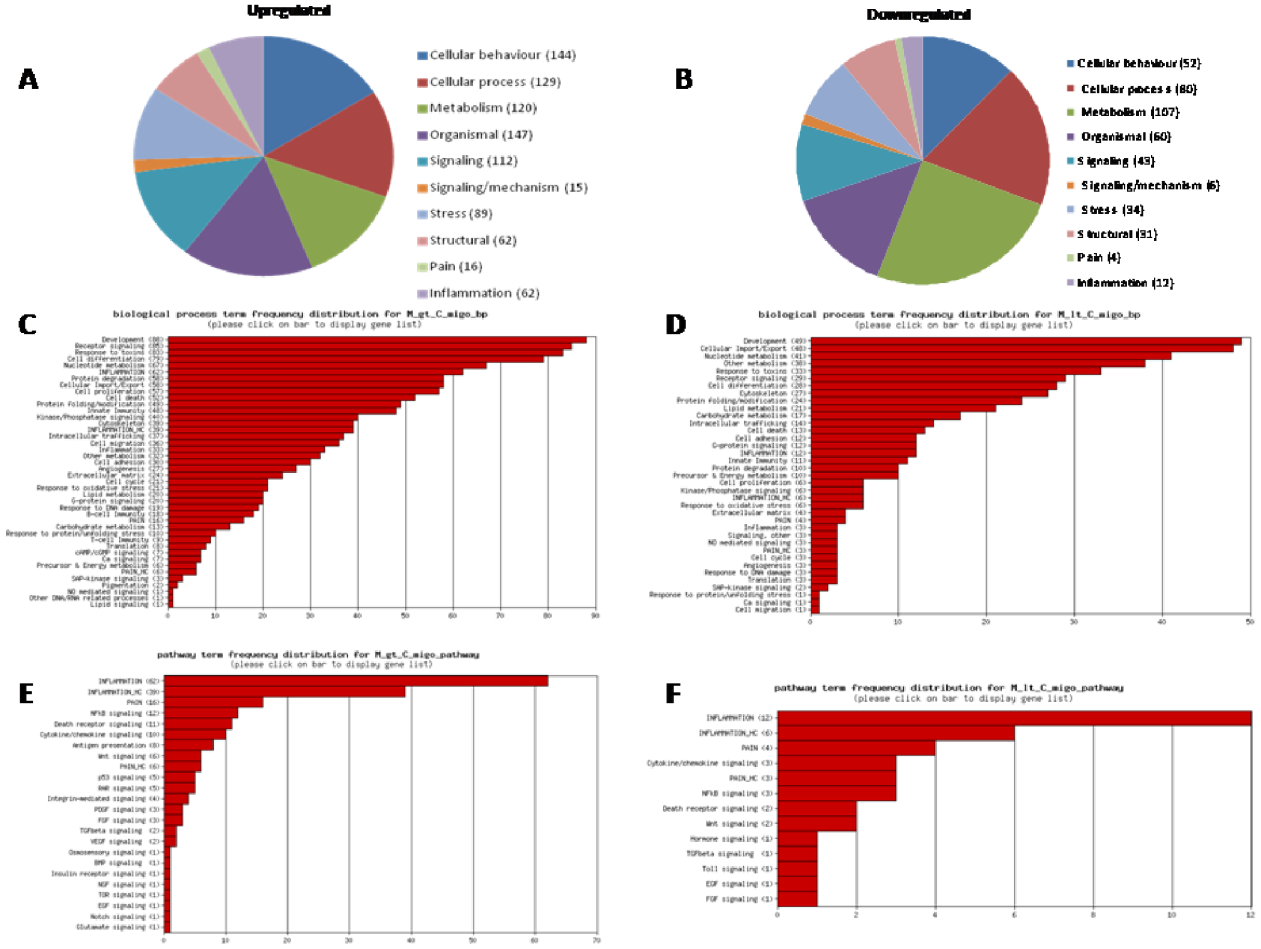
Functional analysis of the effect of VSL#3 on NMS-induced alteration of gene expression. (A–B) Pie charts representing biological processes, molecular functions and cellular components among differentially expressed genes. (C–D) Frequency distribution of the annotations for upregulated and downregulated genes respectively. Note that in animals treated with probiotic diet there were more genes belonging to the annotations “Pain” and “Inflammation” that were downregulated than those upregulated. (E–F) Frequency distribution of the signaling pathways for upregulated and downregulated genes respectively. gt: greater than; lt: lower than; migo_bp: gene ontology (GO) curated by Miltenyi Bioinformatics biological processs/function; migo_pathways: gene ontology (GO) curated by Miltenyi Bioinformatics pathways; Inflammation_HC: list of genes associated with inflammation from GO data but with more stringent selection criteria; Pain_HC: list of genes associated with pain from GO data but with more stringent selection criteria.

In NMS rats, probiotic intervention reset the abnormal gene regulation caused by maternal deprivation. The hierarchical clustering and the heat map (Figure 4A and B for up- and downregulated genes respectively) demonstrated that, when compared with NMS (lanes M5–M8), VSL#3 treated rats (lanes D9–D11) showed a robust change in the expression of 779 genes with 411 and 368 up- or downregulated genes (Table 3). Moreover, VSL#3 administration attenuated changes in the expression of majority of genes: mostly of them were normalized to the level of Group C (lanes C1–C4) (Figure 4A and B). The comparison of regulated gene sets between group M compared to group C and group D compared to group M showed that the probiotic diet reversed or significantly attenuated changes occurring in NMS model. Panel C of Figure 4 shows a VENN diagram of the genes upregulated by maternal separation relatively to healthy animals, in comparison to the set of genes downregulated in group D compared to group M. The number of affected genes was comparable, but the majority of genes (202) was found at the intersection – these gene were upregulated in group M but downregulated in group D. The overlap between gene sets downregulated in samples of NMS group and those upregulated by probiotic diet compared to mother deprivation alone is also very high. Indeed, 139 genes were found at the intersection, indicating that the probiotic intervention also reversed the effects of mother separation on these genes (Figure 4D). The functional analysis conducted in group D in comparison with group M showed an overexpression of 20 genes correlated with “Pain” and “Inflammation” categories, while 79 genes were globally downregulated, demonstrating that VSL#3 treatment was counter-regulatory on gene expression caused by NMS (Figure 5A and B respectively). The frequency distribution of significantly modified genes in representative GO-derivative biological processes/functions (migo_bp) and GO-derivative biological pathways (migo_pathways) for group D in comparison to group M is also illustrated in Figure 5 that shows the signaling pathways in which upregulated (panels C and E) and downregulated (panels D and F) genes were involved. Interestingly, VSL#3 induced downregulation of several gene related to antigen presentation and to immune and inflammatory response. VSL#3 intervention up- or downregulates 8 and 11 genes (TNFRSF1B was identified by two different primers) correlated with “Pain” (Tables 6 and 7 respectively). Among the formers were genes that play a significant role in antinociception, including cannabinoid receptor 1 and opiate receptor-like 1 (Table 6). Moreover, 9 of the 15 genes correlated to “Pain” that were upregulated in NMS rats in comparison with healthy animals were normalized after treatment with VSL#3 (Table 7), indicating that a possible mechanism of action of the probiotic diet was the regulation of genes encoding for protein mediating painful signals. Among others, TPH1, CCL2, CCR2, NOS3, NTRK1, BDKRB1, IL10, TNFRSF1B and TRPV4 genes were significantly downregulated by VSL#3.

**Table 6:** Upregulated genes specifically involved in pain transmission in VSL#3-treated NMS rats in comparison with not treated NMS animals.

Identifier	Short Name	Long Name
A_43_P13083	TACR2	tachykinin receptor 2
A_43_P13418	KCNK2	potassium channel, subfamily K, member 2
A_44_P1030258	CNR1	cannabinoid receptor 1 (brain)
A_44_P166967	NPY1R	neuropeptide Y receptor Y1
A_44_P408520	ENSRNOT00000022509	opiate receptor-like 1
A_44_P472874	TRPA1	transient receptor potential cation channel, subfamily A, member 1
A_64_P003854	IAPP	islet amyloid polypeptide
A_64_P058336	UCN	urocortin

**Table 7:** Downregulated genes specifically involved in pain transmission in VSL#3-treated NMS rats in comparison with not treated NMS animals.

Identifier	Short Name	Long Name
A_42_P695401	CCL2	chemokine (C-C motif) ligand 2
A_42_P714311	CCL3	chemokine (C-C motif) ligand 3
A_44_P198620	NOS3	nitric oxide synthase 3, endothelial cell
A_44_P430547	NTRK1	neurotrophic tyrosine kinase, receptor, type 1
A_64_P033800	SLC1A2	solute carrier family 1 (glial high affinity glutamate transporter), member 2
A_64_P048210	TPH1	tryptophan hydroxylase 1
A_64_P100793	CCR2	chemokine (C-C motif) receptor 2
A_64_P118628	BDKRB1	bradykinin receptor B1
A_64_P122382	IL10	interleukin 10
A_64_P125973	TNFRSF1B	tumor necrosis factor receptor superfamily, member 1b*
A_64_P130174	TRPV4	transient receptor potential cation channel, subfamily V, member 4
A_64_P165297		tumor necrosis factor receptor superfamily, member 1b*

* Two different probe sequences are used for the same gene on the array.

**Figure 6:**
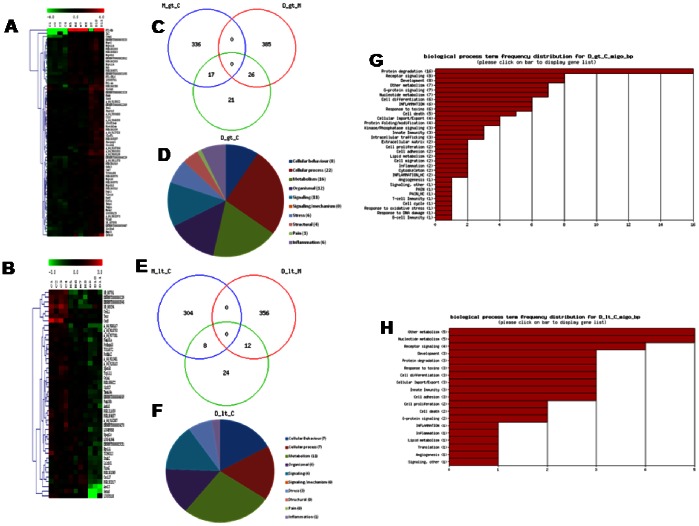
DGA and functional analysis of gene expression of VSL#3-treated NMS rats in comparison with controls. (A–B) Heat maps analysis of changes caused by VSL#3 administration (Group D, lanes D9–D11) to NMS rats on genes whose expression was modulated >2-folds (up- or downregulation, p<0.05) in comparison with control group (Group C, lanes C1–C4). NMS rats treated with VSL#3 showed upregulation and downregulation of 64 and 44 genes respectively, indicating that the probiotic diet resets the colonic expression of genes to values observed in control rats. (C–E) Venn diagrams of regulated genes in Group D in comparison with Group C. In NMS rat, VSL#3 administration induced an upregulation of 411 genes, 26 of which are also upregulated in Group C, and on a total of 368 downregulated genes in Group M, 12 were also dowregulated in Group C. The functional analysis in Group D vs Group C was illustrated by pie charts representing biological processes, molecular functions and cellular components among differentially expressed genes (D and F for up- and downregulated genes respectively). (G–H) Frequency distribution of the annotations for up- and downregulated genes respectively. Note that in VSL#3-treated NMS rats only 1 gene belonging to the category “Pain” (the opiate receptor-like 1 gene) was upregulated and, in generally, a lower number of gene were significantly modified in group D in comparison with group C. gt: greater than; lt: lower than; migo_bp: gene ontology (GO) curated by Miltenyi Bioinformatics biological process/function; Inflammation_HC: list of genes associated with inflammation from GO data but with more stringent selection criteria; Pain_HC: list of genes associated with pain from GO data but with more stringent selection criteria. Microarray data from 3–4 replicates.

When the gene expression driven by administering NMS rats with VSL#3 was compared to that of naïve rats, only a minority of genes were still modified by NMS (Figure 6). Indeed, group D (lanes D9–D11) showed up- and downregulation of 64 and 44 genes respectively in comparison with group C (lanes C1–C4) (Figure 6A and B respectively). Moreover, Venn diagrams demonstrated an overlap of genes that were significantly up- and downregulated among the three groups (Figure 6C and E respectively). The functional GO analysis revealed that only 1 gene belonging to pain annotation was still significantly upregulated in group D in comparison with Group C (Table 8), while no gene was downregulated, demonstrating that VSL#3 treatment in NMS rats induced an almost complete normalization of gene expression. Finally, the functional analysis showed an overexpression of a total of 6 genes correlated with “Inflammation” category, while only 1 gene was globally downregulated (Figure 6D and F respectively). The frequency distribution of significantly modified genes in representative GO-derivative biological processes/functions (migo_bp) conducted in group D in comparison with group C is also reported (Figure 6G and H).

**Table 8:** Upregulated genes specifically involved in pain transmission in VSL#3-treated NMS rats in comparison with normal animals.

Identifier	Short Name	Long Name
A_44_P408520	ENSRNOT00000022509	opiate receptor-like 1

### PCR Analysis

**Figure 7:**
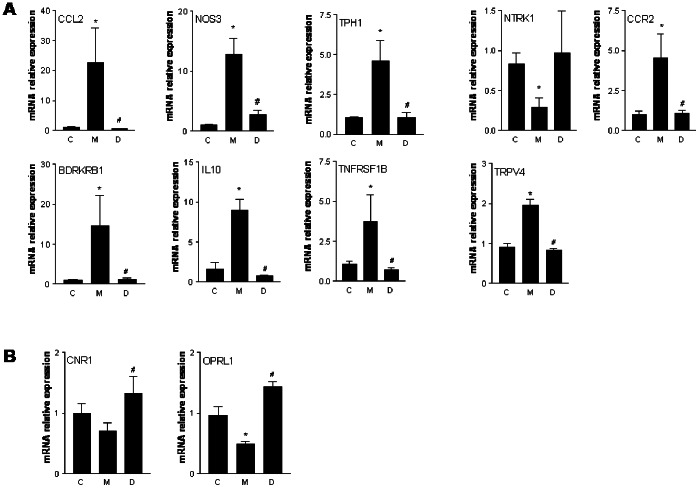
Confirmation of microarray data by qRT-PCR analysis. (A) Colon expression of the “Pain” annotation-related genes that were upregulated in Group M and downregulated in Group D. (B) Gene expression of the “Pain” annotation-related genes that were upregulated in Group D in comparison with Group M. *p<0.05 vs Group C; ^#^p<0.05 vs Group M.

The results of PCR analysis confirmed the microarray data for nociceptive (except for the NTRK1 gene) (Figure 7A) and antinociceptive genes (CNR1 and OLR-1; Figure 7B). *p<0.05 versus Group C; #p<0.05 versus Group M.

### Interaction Network: Core Inflammatory Pathway

**Figure 8:**
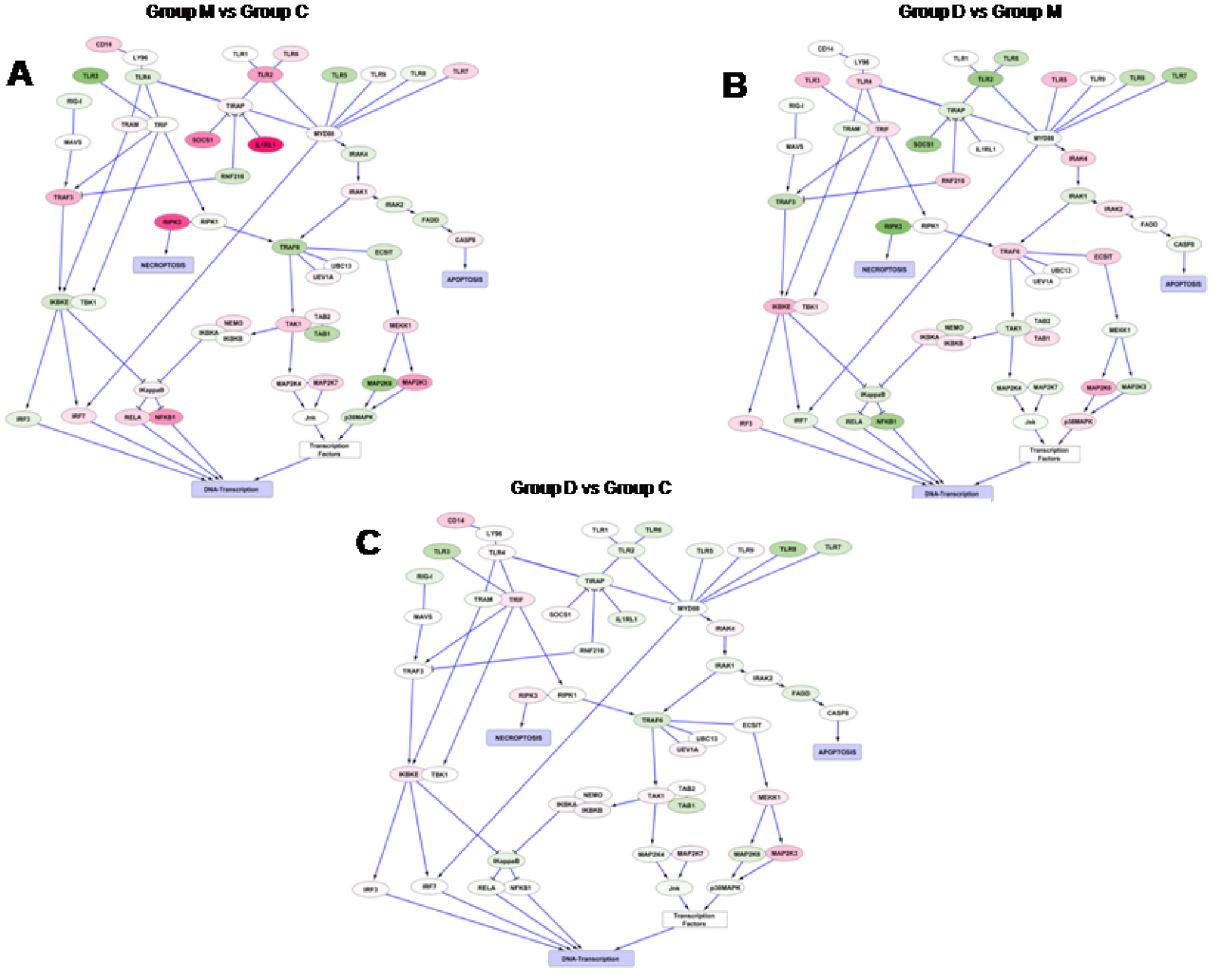
Interaction network: core inflammatory pathway. (A–B) The expression of several genes of the pro-inflammatory pathways which were upregulated (red color) or downregulated (green color) in group M compared with group C was neutralized upon treatment with the probiotic diet. This effect was observed for both receptors expressed on the cell surface and components of various signal transduction cascades including NF kB, IRFs and MAPK pathways. (C) VSL#3 almost completely restored the initial pattern of inflammation-related genes.

The observations made in the previous analyses were confirmed by analysis of the interaction graphs. Many genes of the pro-inflammatory pathways which are upregulated (in red) or downregulated (in green) in the NMS group compared to healthy animals (Figure 8A) were normalized upon additional treatment with probiotic diet (Figure 8B). This effect is observed for both receptors expressed on the cell surface and components of various signal transduction cascades including NFκB, IRFs and MAPK pathways. In contrast, NMS rats administered VSL#3 gained an almost complete normalization of the inflammation-related genes pattern when compared with naive rats (Figure 8C).

## Discussion

In the present study we have shown that the neonatal stress caused by NMS alters the expression of large cohort of genes in the colonic tissue and that these changes might be mechanistically linked to development of pain. Additionally, we have shown that this pattern could be corrected by administering NMS rats with a probiotic diet.

Early stress life events in the form of maternal separation induce permanent alterations in the rat and predispose to develop increased colonic motility and permeability [6] and hypersensitivity to mucosal inflammation in adulthood [37]. Further, maternal deprivation results in permanent changes in the central nervous system including unrestrained secretion of corticotrophin-releasing factor [38], alterations in serotoninergic, noradrenergic and dopaminergic systems [39], decreased expression of benzodiazepine and γ-aminobutyric acid type A receptors [40]. All pathways are potentially involved in development and maintenance of visceral pain.

In the present study we have shown that NMS rats exhibit visceral hyperalgesia and allodynia in response to CRD, indicating that stress in early life induces profound and long-standing changes in the development of the central nervous system and a modification of the neural pathways that result in an increase of pain perception. These changes were attenuated by administering rats with a probiotic mixture VSL#3. The timing of VSL#3 administration, however, had relevance. Indeed, VSL#3 reduces visceral pain not only when administered for the duration of the study, but also when it was administered in the last 2 weeks before CRD. In contrast, when VSL#3 intervention was performed for the first 2 weeks, it reverses allodynia but not hyperalgesia [41].

The effects of VSL#3 on NMS-induced hypersensitivity is not due to a change of the state of consciousness or modification of colorectal tone since VSL#3 administration had no effect on colorectal compliance (see Figure S1).

To elucidate the mechanisms of visceral hypersensitivity, we adopted a microarray approach. Analysis of colonic genetic pattern of NMS rats demonstrated that exposure to a stressful stimulus in the early life altered the expression of approximately one third (28%) of whole genome. However, the combination of a restricted DGA with the functional analysis revealed that less than 3% of genes, belonging to several annotation categories, are significantly up- or downregulated in NMS rats. The larger number of genes was grouped in the “Metabolism” category. This group includes carbohydrate and protein metabolisms. Our data agree with those of Lopes *et al.* who have shown that NMS affects the neuromuscular protein profile of the rat colon, in particular the expression of proteins whose functions are related to purine metabolism, protein folding and carbohydrate metabolism [42].

Although we have shown that NMS influences expression of many genes, we have focused our attention on the “*Pain*” annotation category, finding 15 upregulated and 4 downregulated genes that may have relevance in the development and maintenance of visceral pain in this model. Interestingly, the GO-based functional analyses has revealed also a correlation between “Inflammation” and “Pain” categories, as many genes are included in both annotations.

In the present study, data converge onto the hypothesis that VSL#3 intervention inhibits CRD-induced pain by reversing the alterations of genetic pattern caused by early maternal deprivation, specifically by influencing those pathways involved in pain and inflammation. First, the global genic analysis has demonstrated that VSL#3 induces the change of approximately 30% of the genes in NMS rats in comparison with non treated NMS animals and, more interestingly, the global genic pattern of NMS rats exposed to VSL#3 was similar to that of healthy animals, as only 6% of genes were modified. In other words, the genic pattern of NMS rats treated with probiotic diet was similar to that of naïve animals, as the expression of great majoriry of genes (94%) was similar in the two groups of animals. Second, DGA analysis confirms that VSL#3 reversed the alterations induced by maternal deprivation. Indeed, VSL#3 caused the significant modification of 779 genes in comparison with NMS, not treated rats and the great majority of genes that are significantly up- or downregulated in NMS rats are then counter-regulated by VSL#3 administration. In contrast, only 108 genes are significantly modified in VSL#3-treated NMS animals in comparison with naïve rats. Third, both the functional analysis and the qRT-PCR studies have demonstrated that the expression of several genes related to “Pain” and “Inflammation” annotation categories was counter-regulated by VSL#3 intervention. In particular, TPH1, CCL2, CCR2, NOS3, NTRK1, BDKRB1, IL10, TNFRSF1B and TRPV4, all genes encoding for proteins involved in nociception, are upregulated in NMS, hypersensitive rats and downregulated in NMS animals treated with VSL#3. Among them, TPH1 has a central role in nociception. This gene encodes for the tryptophan hydroxylase 1, an enzyme that catalyzes the first and rate limiting step in the biosynthesis of serotonin. Tryptophan hydroxylase 1 is involved in both neuropathic and visceral pain in animal models of nociception and in humans disorders. Thus, Crohn’s disease patients who experience IBS-like symptoms are characterized by increased expression of tryptophan hydroxylase 1 in the colon [43]. Further, inhibition of tryptophan hydroxylase 1 in the gastrointestinal tract in IBS patients reduces mucosal production of serotonin and ameliorates symptoms [44]–[45]. A phase 2 clinical trial with the tryptophan hydroxylase 1 inhibitor LX1031 in patients with non-constipating IBS has been recently reported [46]. Fourth, exposure to VSL#3 upregulates the expression of several genes related to induction of analgesia, as cannabinoid receptor 1 (brain) (CNR1) [16], [47] and opiate receptor-like 1 (ENSRNOT0000002250), although the role of the latter gene in both nociception and analgesia is still controversial [48]–[49]. These findings agree with previous observations indicating that probiotics induce the expression of δ-opioid and cannabinoid receptors in intestinal epithelial cells, mediating analgesic functions in the gut in a way that mirrors the effect of morphine [16]. Fifth, It has been demonstrated recently that VSL#3 up- or downregulates the expression of several genes related to immunomodulation and inflammation [50], including the expression of the proinflammatory chemokine MCP-1/CCL2 in macrophages of children affected by Crohn disease [51]. Moreover, VSL#3 corrects the inflammation-driven dysregulation of PPARγ, FXR and leptin in a rodent model of inflammation [52]. Present findings are consistent with these observation indicating that VSL#3 counter-regulates genes involved in the inflammatory cascade, including CCL2, NOS3, IL10 and TNFRSF1B and genes that encode for factors that regulate the innate and adaptive immune response, as TLRs, NFκB and MAPKs, thus inhibiting inflammatory and, indirectly, nociceptive processes.

In conclusion, this report illustrates a novel approach to the analysis of the mechanisms underlying the pathogenesis of pain in experimental model of IBS. This approach allowed the identification of novel regulatory mechanisms and specific patterns of genic expression caused by exposure to probiotic that might have clinical readouts in conditions of visceral pain and stress-correlated intestinal pathologies.

## Supporting Information

Figure S1 (A) Group C and Group M are the same in all comparison experiments. (A) Maternal deprivation and (B, C, D) VSL#3 intervention performed in different periods had any effect on colorectal compliance.
(TIF)
