# Supplementary figures and images for: Probiotics VSL#3 Protect against Development of Visceral Pain in Murine Model of Irritable Bowel Syndrome
Source: PLoS One. 2013 May 15;8(5):e63893. doi: 10.1371/journal.pone.0063893 (PMC3655059; doi:10.1371/journal.pone.0063893)

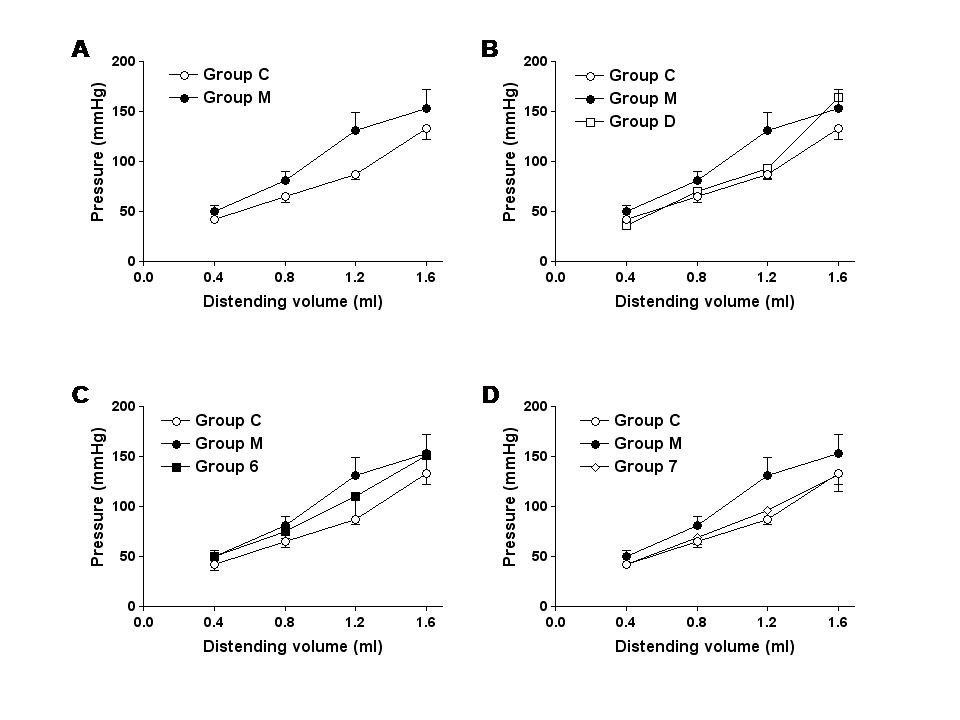

Supplement: Figure S1 — (A) Group C and Group M are the same in all comparison experiments. (A) Maternal deprivation and (B, C, D) VSL#3 intervention performed in different periods had any effect on colorectal compliance. (TIF) [file pone.0063893.s001.tif]
